# Supplementary material for: REM sleep promotes experience-dependent dendritic spine elimination in the mouse cortex
Source: Nat Commun. 2020 Sep 23;11:4819. doi: 10.1038/s41467-020-18592-5 (PMC7511313; doi:10.1038/s41467-020-18592-5)
Supplement: Supplementary file 1 — Supplementary Information [file 41467_2020_18592_MOESM1_ESM.pdf]

Supplementary Information

REM sleep promotes experience-dependent dendritic spine elimination in the mouse cortex

Zhou et al.

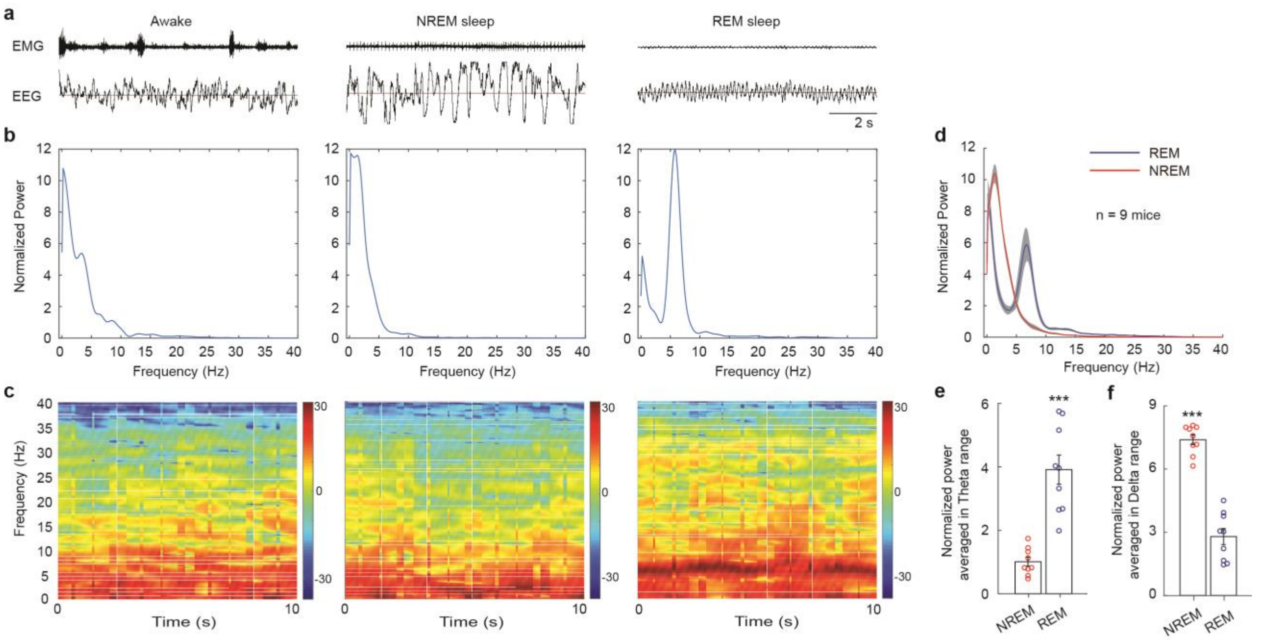

Supplementary Figure 1

Various brain states can be distinguished by EEG/EMG recording.

(a-c) Representative examples of EMG and EEG traces (a) and their relative EEG normalized power (b) and spectrograms (c) in various brain states, respectively. Scale bar: 2 s. (d) Normalized EEG power during NREM sleep (red) and REM sleep (blue) states, mean ± s.e.m (gray envelope) (n = 9 mice). (e-f) Normalized EEG power averaged in theta range (e) (n = 9 mice, $P < 0.0001$) and delta range (f) (n = 9 mice, $P < 0.0001$) (Wilcoxon-Mann-Whitney test, two-sided). *** $P < 0.001$. Data are presented as mean ± s.e.m. Source data are provided as a Source Data file.

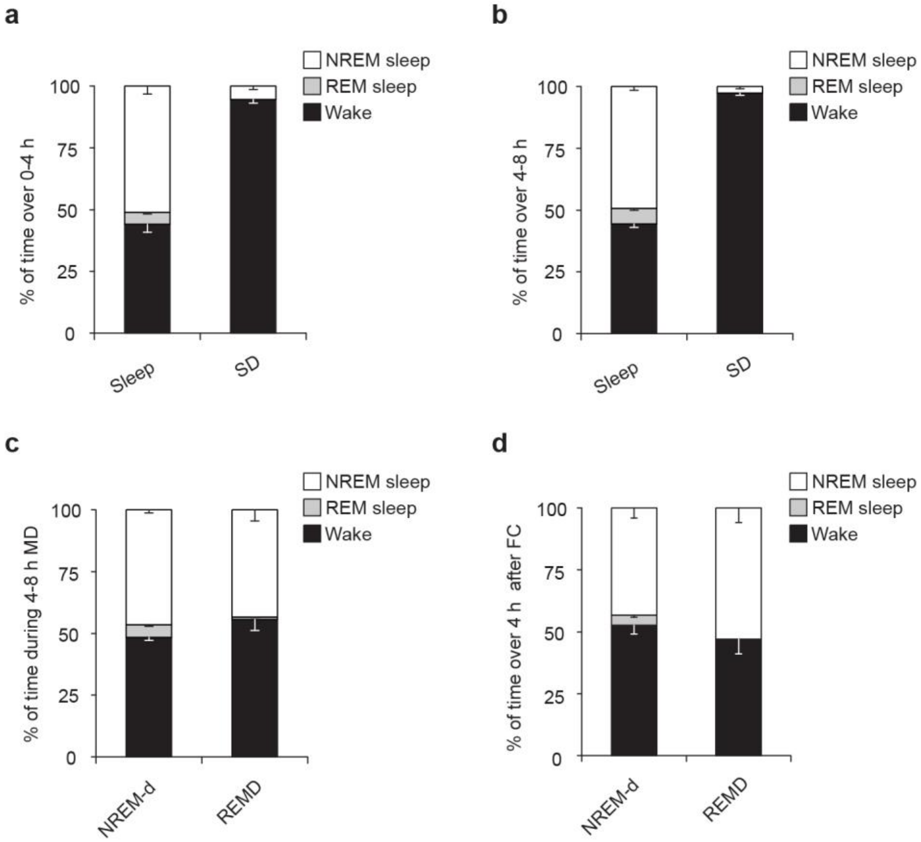

Supplementary Figure 2

SD or REMD by gentle handling reduces the amount of sleep or REM sleep over 4 hours.

(a) Sleep structure of 0–4 hours in undisturbed sleep and SD animals (n = 8 sleep mice and n = 4 SD mice, P = 0.0040 for awake time in sleep and SD animals, Wilcoxon-Mann-Whitney test, two-sided). (b) Sleep structure of 4–8 hours in undisturbed sleep and SD animals (n = 6 sleep mice and n = 6 SD mice, P = 0.0022 for awake time in sleep and SD animals, Wilcoxon-Mann-Whitney test, two-sided). (c, d) Sleep structure in NREM-d and REMD animals. The amount of REM sleep over 4 hours was significantly reduced in REMD mice as compared to NREM-d mice in experiments related to MD (c) (n = 6 NREM-d mice and 6 REMD mice, P = 0.0022 for REM sleep time in NREM-d and REMD animals) and FC (d) (n = 4 NREM-d mice and 4 REMD mice, P = 0.0286 for REM sleep time in NREM-d and REMD animals) (Wilcoxon-Mann-Whitney test, two-sided). Data are presented as mean ± s.e.m. Source data are provided as a Source Data file.

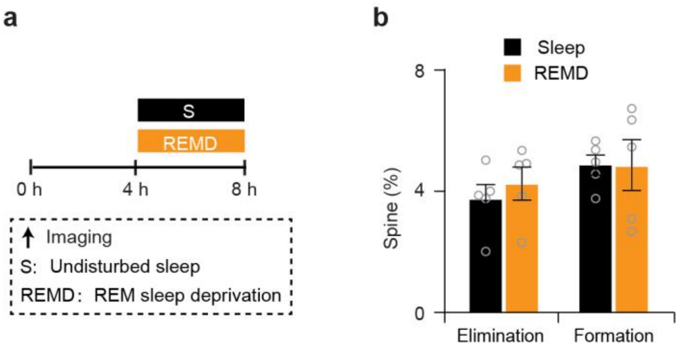

Supplementary Figure 3

REMD over 4–8 hours has no significant effect on dendritic spine remodeling in mice without MD.

(a) Schematic of experimental design to test the effect of REM sleep in control animals without MD. (b) REMD had no significant effect on either dendritic spine elimination or formation in the second 4 h in control animals without MD (n = 5 sleep mice and n = 5 REMD mice, $P = 0.465$ for elimination and $P = 0.754$ for formation, Wilcoxon-Mann-Whitney test, two-sided). Data are presented as mean ± s.e.m. Source data are provided as a Source Data file.

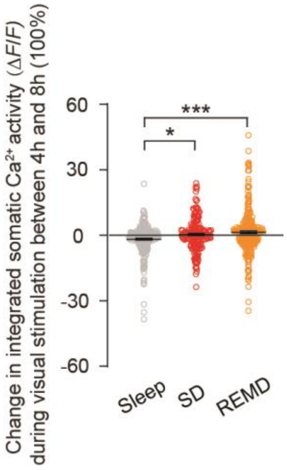

Supplementary Figure 4

Integrated somatic Ca^{2+} activity induced by MD in mice with sleep is significantly lower than that in mice with SD or REMD.

The integrated somatic Ca^{2+} activity of layer 5 pyramidal neurons during visual stimulation showed significant decrease in mice with an additional MD and undisturbed sleep in the second 4-h period when compared to mice with SD or REMD (n = 250 cells from 4 mice, 194 cells from 4 mice and 294 cells from 3 mice for sleep, SD and REMD, respectively; $P = 0.0004$, Kruskal-Wallis test followed by multiple comparisons test). * $P < 0.05$, *** $P < 0.001$. Data are presented as mean ± s.e.m. Source data are provided as a Source Data file.

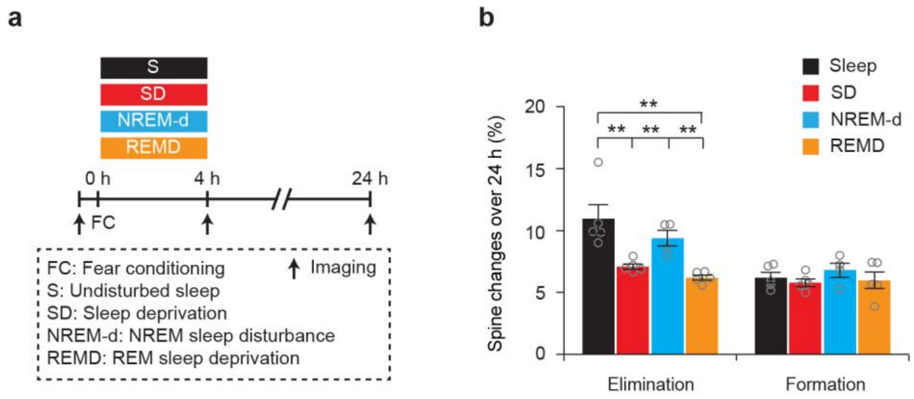

Supplementary Figure 5

SD or REMD over 0–4 hours significantly reduces FC-induced dendritic spine elimination over 24 hours.

(a) Schematic of experimental design to test the effect of sleep or REM sleep on FC-induced dendritic spine remodeling over 24 hours. (b) SD or REMD over 0–4 hours significantly reduced FC-induced dendritic spine elimination. There was no significant difference among all groups in spine formation over 24 hours. (Sleep: 740 spines from 5 animals; SD: 835 spines from 5 animals; REMD: 989 spines from 5 animals; NREM-d: 567 spines from 4 animals; $P = 0.0015$ for elimination and $P = 0.6326$ for formation, Kruskal-Wallis test followed by multiple comparisons test). ** $P < 0.01$. Data are presented as mean ± s.e.m. Source data are provided as a Source Data file.

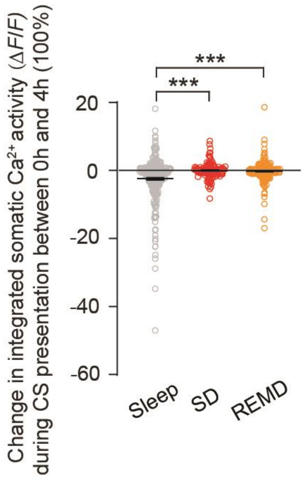

Supplementary Figure 6

Integrated somatic Ca^{2+} activity after FC in mice with sleep is significantly lower than that in mice with SD or REMD.

As compared to mice with SD or REMD over 4 hours, the integrated somatic Ca^{2+} activity of layer 5 pyramidal neurons showed a significant reduction during CS presentation after FC (n = 309 cells from 4 mice, 122 cells from 5 mice and 195 cells from 3 mice for sleep, SD and REMD, respectively; $P < 0.0001$ for sleep vs. SD and REMD, respectively, Kruskal-Wallis test followed by multiple comparisons test). *** $P < 0.001$. Data are presented as mean ± s.e.m. Source data are provided as a Source Data file.

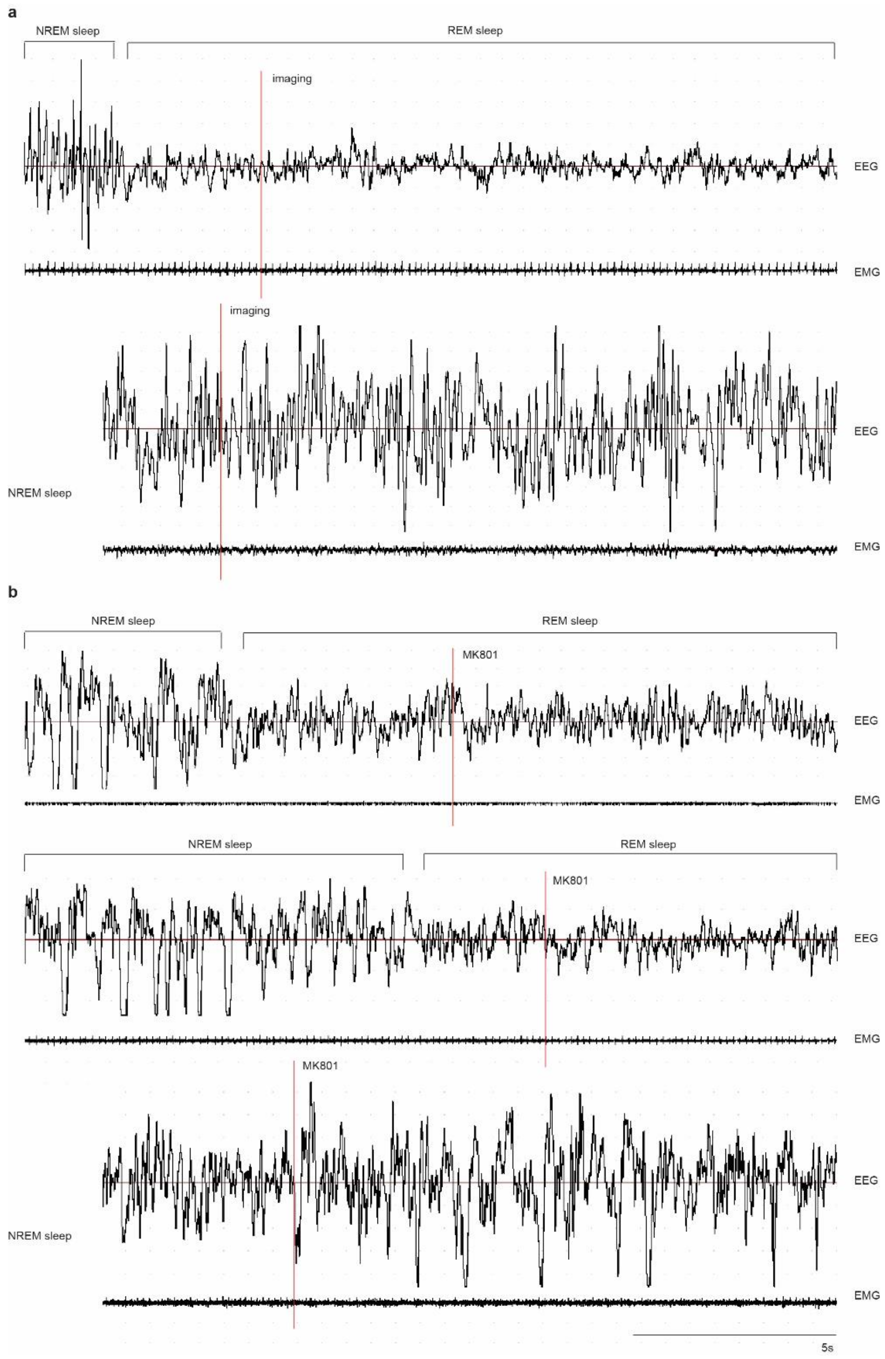

Supplementary Figure 7

Two-photon Ca^{2+} imaging or injection of MK801 procedure has no effect on the EEG and EMG patterns during NREM and REM sleep in head-restrained animals.

EEG and EMG recordings showed that sleep patterns were not interrupted by the procedures of two-photon imaging (a) or injection of MK801 (b). Red lines indicated the time of two-photon scanning or MK801 injection.

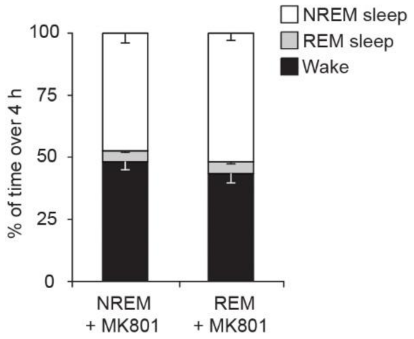

Supplementary Figure 8

MK801 injection has no effect on the sleep structure in head-restrained animals.

Sleep structure in head-restrained animals subjected to MK801 injection during NREM sleep (n = 5 mice) and REM sleep (n = 4 mice), respectively (NREM + MK801 vs. REM + MK801 vs. sleep group shown in Supplementary Figure 2a: P = 0.8502, = 0.7766 and = 0.7392 for Wake, NREM sleep and REM sleep, respectively, Kruskal-Wallis test followed by multiple comparisons test). Data are presented as mean ± s.e.m. Source data are provided as a Source Data file.

.
