# Supplementary material for: REM sleep promotes experience-dependent dendritic spine elimination in the mouse cortex
Source: Nat Commun. 2020 Sep 23;11:4819. doi: 10.1038/s41467-020-18592-5 (PMC7511313; doi:10.1038/s41467-020-18592-5)
Supplement: Supplementary file 3 — Description of Additional Supplementary Files [file 41467_2020_18592_MOESM3_ESM.pdf]

Description of Additional Supplementary Files

File Name: Supplementary Movie 1
Description: 3-D image stacks of dendrites corresponding to 2-D images at 0 h in control animal.

File Name: Supplementary Movie 2
Description: 3-D image stacks of dendrites corresponding to 2-D images at 24 h in control animal.

File Name: Supplementary Movie 3
Description: 3-D image stacks of dendrites corresponding to 2-D images at 0 h in MD animal.

File Name: Supplementary Movie 4
Description: 3-D image stacks of dendrites corresponding to 2-D images at 24 h in MD animal.

File Name: Supplementary Movie 5
Description: **Ca2+ imaging of apical tuft dendrites of layer 5 pyramidal neurons expressing the genetically encoded Ca2+ indicator GCaMP6s in V1 under various brain states.** Two-photon Ca2+ imaging of apical tuft dendrites of layer 5 pyramidal neurons in V1 in head-restrained mice. The video showed dendritic Ca2+ spikes over a period of ~40 seconds under quiet awake, NREM sleep and REM sleep, respectively. Scale bar, 10 µm. 3 circles showed small and bright fluorescent structures as references to make sure that the same focal plane was imaged across various states.

File Name: Supplementary Movie 6
Description: **Ca2+ imaging of apical tuft dendrites of layer 5 pyramidal neurons expressing the genetically encoded Ca2+ indicator GCaMP6s in FrA under various brain states.** Two-photon Ca2+ imaging of apical tuft dendrites of layer 5 pyramidal neurons in FrA in head-restrained mice. The video showed dendritic Ca2+ spikes over a period of ~40 seconds under quiet awake, NREM sleep and REM sleep, respectively. Scale bar, 10 µm. 3 circles showed small and bright fluorescent structures as references to make sure that the same focal plane was imaged across various states.
